# Lichen Scrofulosorum Without Mantoux Positivity in a 72-Year-Old Male: A Clinical Paradox

**DOI:** 10.7759/cureus.99689

**Published:** 2025-12-20

**Authors:** Jebisha Joseph Bella, Manu Vidhya Harikumar, Leena Dennis Joseph, Sudha Rangarajan, Adikrishnan Swaminathan

**Affiliations:** 1 Department of Dermatology, Venereology and Leprosy, Sri Ramachandra Institute of Higher Education and Research, Chennai, IND; 2 Department of General Pathology, Sri Ramachandra Institute of Higher Education and Research, Chennai, IND

**Keywords:** granulomatous inflammation, lichen scrofulosorum, mantoux negativity, tuberculid, tuberculous lymphadenopathy

## Abstract

Lichen scrofulosorum (LS) represents a cutaneous hypersensitivity reaction to Mycobacterium tuberculosis (MTB) in individuals with preserved cell-mediated immunity. It typically presents as asymptomatic, monomorphic papules, predominantly distributed over the trunk, and demonstrates a prompt response to anti-tubercular pharmacotherapy. While the Mantoux test is usually strongly positive, this case highlights the atypical occurrence of Mantoux negativity in a 72-year-old patient, an age group in which LS is rarely encountered. This case emphasizes the need for a comprehensive diagnostic workup, including histopathological analysis and other relevant investigations, when the Mantoux test yields an unexpected result. A keen clinical eye and strong diagnostic suspicion are essential to identifying LS in the context of such atypical features.

## Introduction

Lichen scrofulosorum (LS) is a rare cutaneous manifestation of tuberculosis, categorized as a tuberculid [1]. Although the exact pathogenesis is not fully elucidated, it is hypothesized to result from a hypersensitivity reaction to the hematogenous dissemination of Mycobacterium tuberculosis (MTB) antigens from a distant focus [2]. LS is typically associated with a strongly positive tuberculin skin test and is most commonly reported in children and adolescents, reflecting an immunologically active host response [1]. We report an uncommon presentation of LS in a 72-year-old male with a negative Mantoux test, which posed a diagnostic challenge due to its atypical nature. The presence of cervical lymphadenopathy, subsequently confirmed to be of tuberculous origin, along with the resolution of skin lesions following antitubercular therapy, played a crucial role in establishing the final diagnosis.

## Case presentation

A 72-year-old man presented to the outpatient department with a four-month history of multiple, grouped, raised skin-coloured tiny lesions over the bilateral upper limbs, trunk, and bilateral lower limbs. The skin lesions were asymptomatic but occasionally pruritic. The patient reported a history of losing six kilograms of weight over the past eight months. There was no history of cough, evening rise of temperature, or drug intake prior to the onset of the lesions.

**Figure 1:**
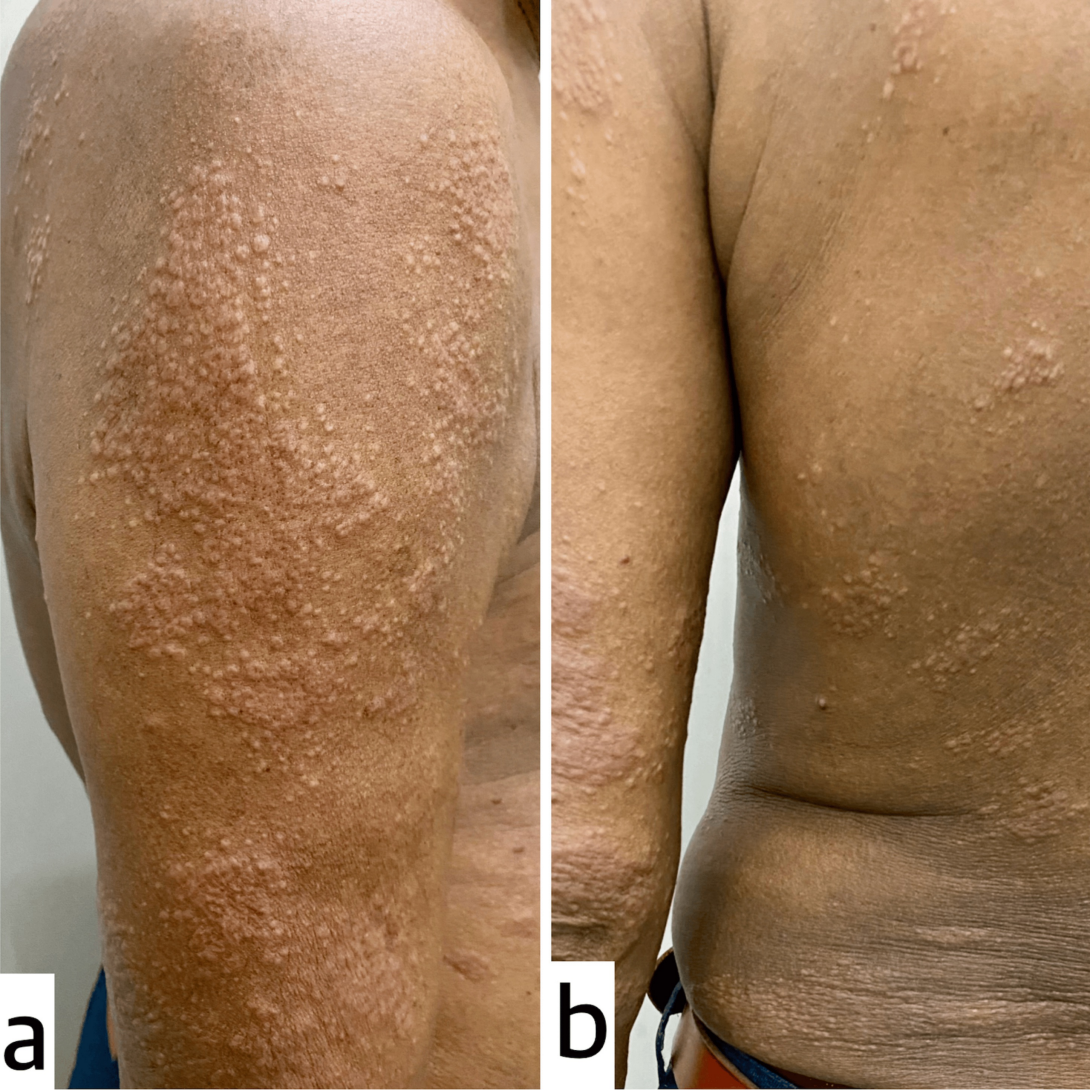
Multiple skin-colored to erythematous, monomorphic follicular papules coalescing to form plaques observed over the right upper limb (a) and left lower back (b).

Cutaneous examination revealed multiple skin-coloured, follicular, monomorphic papules of size 1-3 mm coalescing to form plaques distributed over bilateral upper limbs, trunk and bilateral lower limbs (Figure 1a, 1b). Firm, matted and tender lymphadenopathy of size 2x3 cm was palpated in the right supraclavicular region. Differential diagnosis of papular sarcoidosis, secondary syphilis and mucinosis was considered.

Laboratory investigations showed a hemoglobin level of 10.3 g/dL. Angiotensin-converting enzyme (ACE) level was within normal limits, and sarcoidosis was ruled out. A summary of the laboratory findings with corresponding reference ranges is presented (Table 1). Viral serologies were non-reactive (Table 2). Chest radiography showed bilateral clear lung fields.

**Table 1:** Laboratory findings

Parameter	Result	Reference value	Unit
Haemoglobin	10.3	13-17	g/dL
Total leukocyte count	5770	4000-11000	cells/cubic mm
Erythrocyte Sedimentation Rate (ESR)	107	4-30	mm/hr
Lactate Dehydrogenase (LDH)	143	135-225	U/L
Fasting plasma glucose	102	70-110	mg/dL
Post-prandial plasma glucose	112	80-140	mg/dL
Angiotensin Converting Enzyme (ACE)	22	13.3-63.9	U/L
Calcium	8.8	8.8-10.2	mg/dL
C-Reactive Protein	1.97	0-0.8	mg/dL

**Table 2:** Serological test results

Test	Result
Human Immunodeficiency Virus P24 antigen (HIV P24)	Non-reactive
Human Immunodeficiency Virus-I & II antibodies (HIV I & II Ab)	Non-reactive
Hepatitis B surface antigen (HBsAg)	Non-reactive
Hepatitis C Virus antibody (HCV Ab)	Non-reactive
Venereal Disease Research Laboratory test (VDRL)	Non-reactive

**Figure 2:**
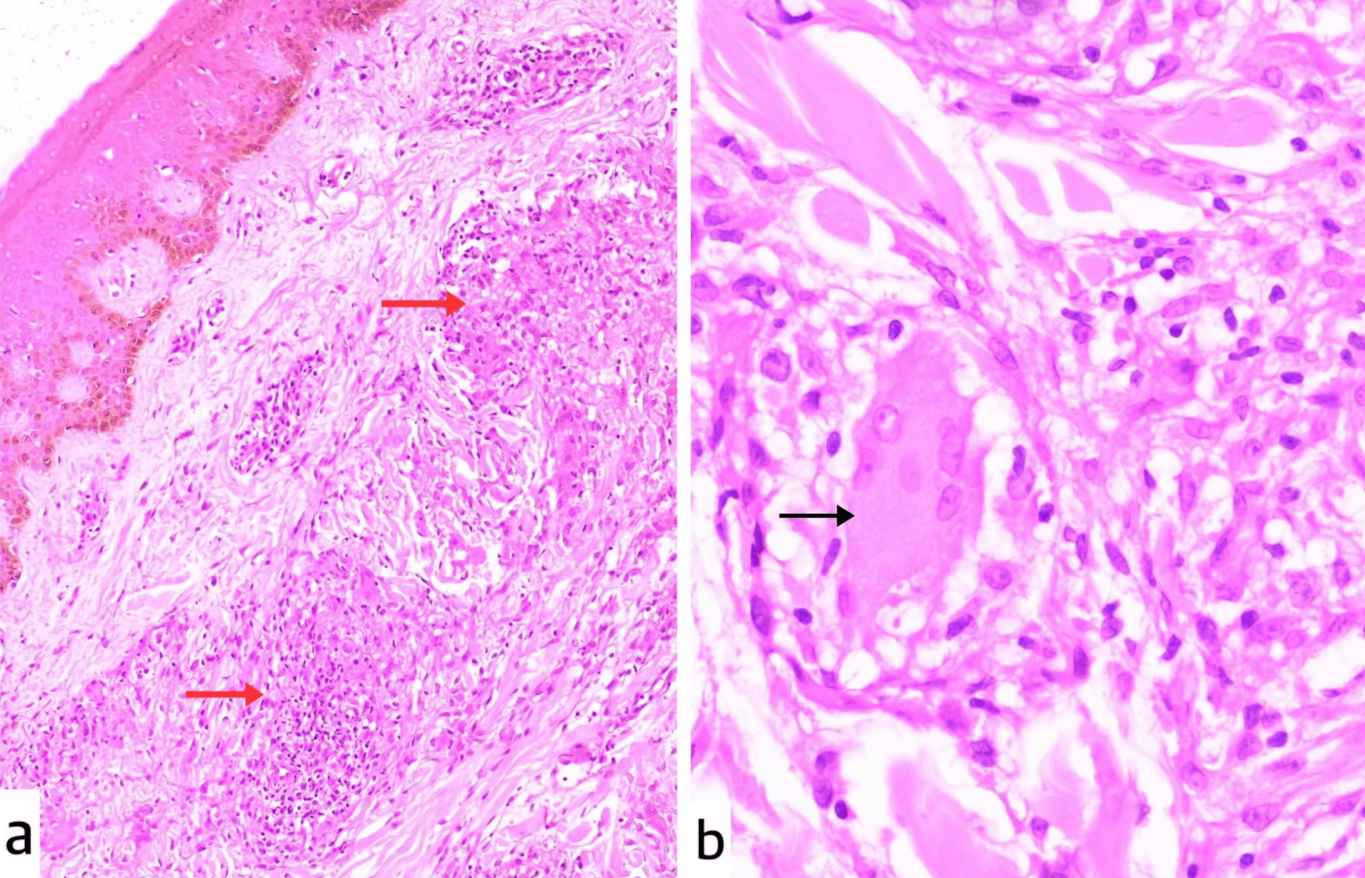
Histopathological examination of a skin punch biopsy from the right upper limb, stained with hematoxylin and eosin (H&E), showing perivascular lymphocytic infiltrates in the upper dermis and epithelioid granulomas (red arrows) in the mid-dermis at 40× magnification (a). Biopsy from the lower back shows Langhans giant cells (black arrow) at 100× magnification (b).

**Figure 3:**
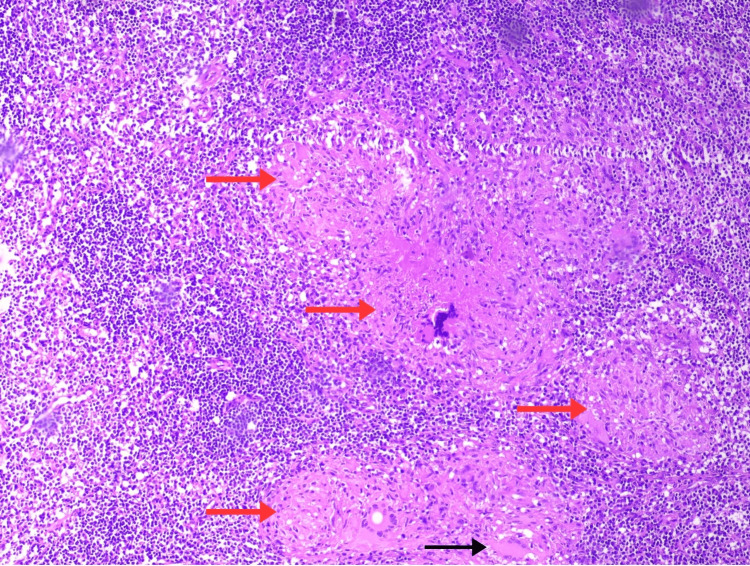
Histopathological examination of a lymph node, stained with hematoxylin and eosin (H&E), showing epithelioid granulomas (red arrows), Langhans giant cells (black arrow), and areas of necrosis at 40× magnification.

Skin punch biopsies from the right arm and lower back revealed epithelioid granuloma in the upper and deep dermis with Langhans giant cells (Figure 2). Diagnosis of tuberculid was suspected. Mantoux test was performed and was negative, with two mm induration. Under general anaesthesia, a 2 × 1 cm lymph node from the right supraclavicular region was excised and sent for histopathological study, acid-fast bacilli (AFB) culture and GeneXpert. Histopathology of the lymph node showed epithelioid granulomas with multinucleate giant cell formation and areas of necrosis and haemorrhage (Figure 3). AFB culture did not show any growth; however, GeneXpert detected MTB.

**Figure 4:**
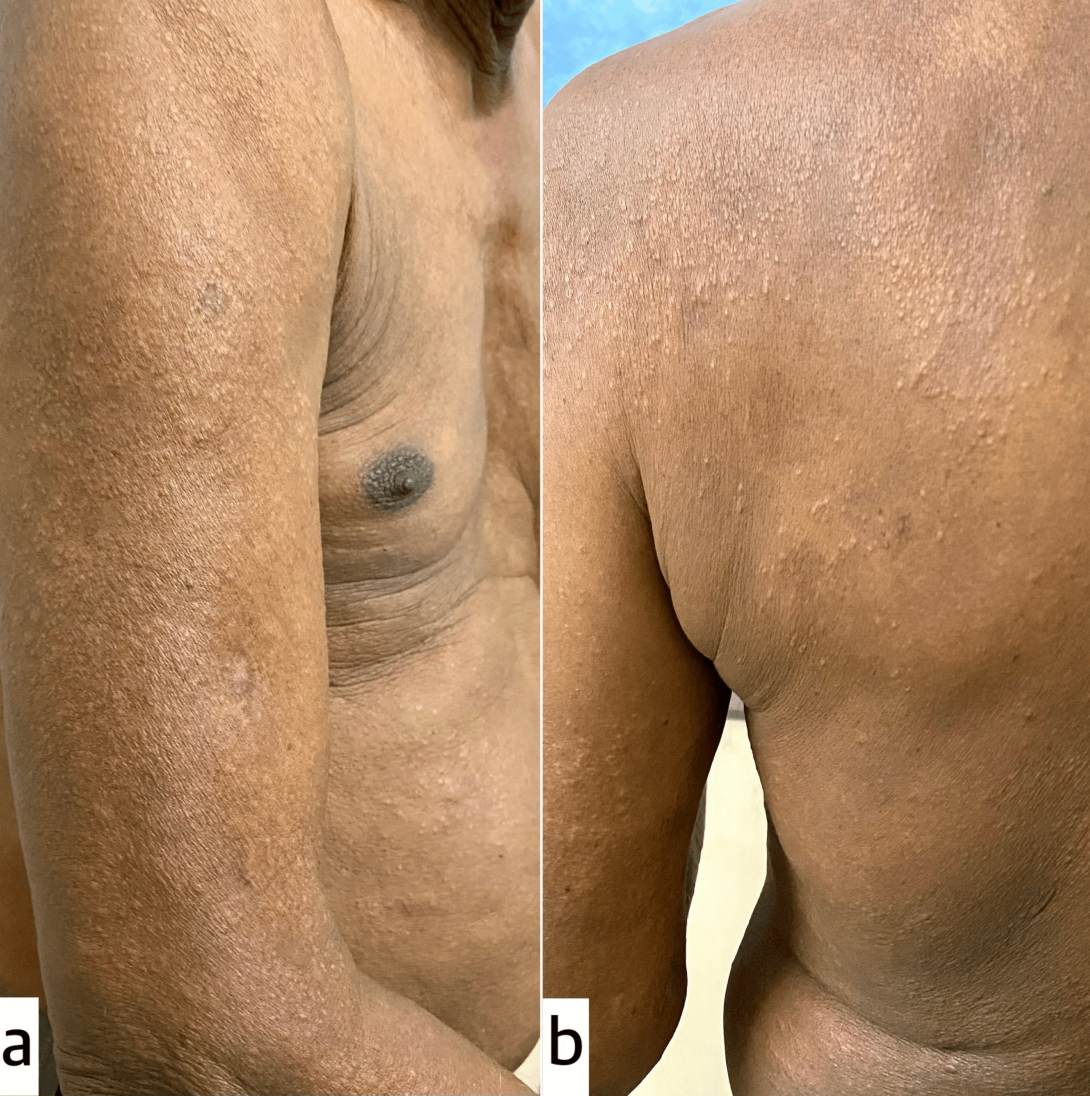
One-month post-treatment follow-up showing marked reduction in the number and size of papules, with flattening of plaques over the right upper limb (a) and left lower back (b) after one month of anti-tubercular therapy (ATT).

A final diagnosis of LS was made. The patient was started on first-line anti-tubercular treatment (ATT) drugs, including Rifampicin, Isoniazid, Pyrazinamide, and Ethambutol under the directly observed treatment short course (DOTS) program. The skin lesions began resolving, and the patient started gaining weight within one month of treatment (Figure 4a, 4b).

## Discussion

LS, also termed tuberculosis cutis lichenoides, is an uncommon cutaneous manifestation of tuberculosis classified under the spectrum of tuberculids [3]. Although the exact pathophysiologic mechanism remains incompletely understood, prevailing hypotheses suggest that LS arises in individuals with tuberculin sensitivity, following hematogenous dissemination of mycobacterial antigens from an often clinically silent tuberculous focus. This antigenic exposure is believed to initiate an immune cascade beginning with immune complex formation, consistent with a type III hypersensitivity response. Over time, persistent antigenic stimulation may promote a subsequent type IV delayed-type hypersensitivity reaction, ultimately leading to the formation of non-caseating granulomas in the dermis [4].

LS is more commonly encountered in children and adolescents, with only five percent of reported cases seen in individuals over the age of 20. This striking age-related pattern may be explained by the physiological decline in delayed-type hypersensitivity responses with advancing age, thereby reducing the likelihood of LS in older individuals [3,5]. The age-related deviation observed in the present case contributed significantly to diagnostic uncertainty.

Considering the rarity of LS beyond adolescence, its diagnosis often depends on a multifaceted approach. This includes a combination of histopathological evidence of granulomatous inflammation, clinical or historical findings of tuberculosis at another site, a typically strong Mantoux test, absence of demonstrable MTB on smear and culture, and a favourable therapeutic response to anti-tubercular treatment. A positive Mantoux test is reported in approximately 83.2% of LS cases. This finding further reinforces the immunological basis of the disease [3]. However, the remaining percentage of cases with a negative Mantoux result cannot be overlooked. These instances raise the possibility that additional or alternative immunopathological mechanisms may be involved in the development of LS, beyond the traditionally postulated hypersensitivity response.

The Mantoux negativity posed a clinical paradox in this case, given that LS is commonly associated with a positive tuberculin skin test. Mantoux negativity is observed in states of immunodeficiency or immune suppression [6]. However, no such underlying immunosuppressive condition was identified in this case. Despite the negative tuberculin skin test, skin and lymph node biopsies demonstrated well-formed epithelioid granulomas with Langhans giant cells, indicating preserved cell-mediated immunity. This contradiction raises important considerations regarding the sensitivity and specificity of the Mantoux test in elderly populations.

Although a negative Mantoux test might have discouraged further evaluation for tuberculosis in a less suspicious clinical setting, persistent clinical suspicion in our patient, based on characteristic cutaneous findings, constitutional symptoms, significant lymphadenopathy, and granulomatous inflammation on histopathology, prompted further investigation. Consequently, a lymph node biopsy with molecular testing using GeneXpert was pursued, which confirmed the presence of MTB and established the definitive diagnosis. The patient’s rapid clinical improvement following initiation of ATT further substantiated the diagnosis.

## Conclusions

This case emphasises the importance of maintaining a high index of suspicion for LS in patients presenting with compatible skin lesions, irrespective of age and Mantoux status. A stepwise diagnostic approach integrating clinical findings, histopathological evidence of granulomatous inflammation, and molecular confirmation of MTB allowed accurate diagnosis despite Mantoux negativity, which might otherwise have led to diagnostic delay. Furthermore, this case invites consideration of additional contributory mechanisms in the pathogenesis of LS beyond the traditionally accepted hypersensitivity reaction to MTB antigens. This case highlights the limitations of relying solely on the tuberculin skin test and underscores the value of multimodal diagnostic strategies in atypical presentations.
